# Inhibition of rice germination by ustiloxin A involves alteration in carbon metabolism and amino acid utilization

**DOI:** 10.3389/fpls.2023.1168985

**Published:** 2023-05-08

**Authors:** Xiaoxiang Fu, Yu Jin, Matthew J. Paul, Minxuan Yuan, Xingwei Liang, Ruqiang Cui, Yingjin Huang, Wenwen Peng, Xiaogui Liang

**Affiliations:** ^1^ The Laboratory for Phytochemistry and Botanical Pesticides, College of Agriculture, Jiangxi Agricultural University, Nanchang, China; ^2^ Key Laboratory of Crop Physiology, Ecology and Genetic Breeding, Ministry of Education/Jiangxi Province, Jiangxi Agricultural University, Nanchang, China; ^3^ College of Agronomy and Biotechnology, China Agricultural University, Beijing, China; ^4^ Plant Sciences, Rothamsted Research, Harpenden, United Kingdom

**Keywords:** seed germination, carbon metabolism, ustiloxins, rice false smut, sugar transport

## Abstract

Ustiloxins are the main mycotoxin in rice false smut, a devastating disease caused by *Ustilaginoidea virens*. A typical phytotoxicity of ustiloxins is strong inhibition of seed germination, but the physiological mechanism is not clear. Here, we show that the inhibition of rice germination by ustiloxin A (UA) is dose-dependent. The sugar availability in UA-treated embryo was lower while the starch residue in endosperm was higher. The transcripts and metabolites responsive to typical UA treatment were investigated. The expression of several SWEET genes responsible for sugar transport in embryo was down-regulated by UA. Glycolysis and pentose phosphate processes in embryo were transcriptionally repressed. Most of the amino acids detected in endosperm and embryo were variously decreased. Ribosomal RNAs for growth were inhibited while the secondary metabolite salicylic acid was also decreased under UA. Hence, we propose that the inhibition of seed germination by UA involves the block of sugar transport from endosperm to embryo, leading to altered carbon metabolism and amino acid utilization in rice plants. Our analysis provides a framework for understanding of the molecular mechanisms of ustiloxins on rice growth and in pathogen infection.

## Highlight

Ustiloxins, the main toxin produced when *Ustilaginoidea virens* infects rice panicles, contaminates rice seeds and inhibits seed germination by inhibiting carbohydrate supply from endosperm to embryo.

## Introduction

With the popularization of hybrids and excessive nitrogen application as well as climate change, rice false smut (RFS) caused by *Ustilaginoidea virens* has become one of the most devastating diseases in rice producing areas such as south and east Asia, the Middle East and North America (Fan et al., 2016; Sun et al., 2020). In China, according to statistics, the average annual area of occurrence and prevention of RFS is 3.06 and 6.92 million ha, resulting in an annual yield loss of 158.6 million kilograms (Lu et al., 2018; Sun et al., 2020). In India, the disease has been reported to occur with an incidence ranging from 5% to 85%, causing yield losses of up to 49% (Dodan and Singh, 1996; Ladhalakshmi et al., 2012). The only visible symptom of RFS is the formation of rice false smut ball (FSB). RFS leads to not only huge yield loss but also serious mycotoxin contamination (Tanaka et al., 2008; Yong et al., 2018; Fan et al., 2020). Ustiloxins, with both animal toxicity and phytotoxicity, are currently known as the main mycotoxin produced during RFS infection and FSB formation (Zhou et al., 2012; Wang et al., 2019). Mycotoxins are important pathogenic factors of plant fungi. For example, the deoxynivalenol (DON), which is a virulence factor for *Fusarium* head blight in wheat, is necessary for fungal spread into floral tissue (Kazan and Gardiner, 2018; Doppler et al., 2019). But for ustiloxins, the potential roles and underlying mechanisms in plant growth and pathogen infection are largely unknown.

Currently, six ustiloxins (A-D, F, and G) have been identified, all of which are water-soluble cyclopeptides containing a 13-membered cyclic core with ether linkage and chiral alkyl (Koiso et al., 1992; Koiso et al., 1998; Wang et al., 2017). Nearly 80% of the ustiloxins are A type (UA) (Shan et al., 2013). FSB can be structurally dissected into inner layer (white endosperm or pseudoparenchyma), middle layer (mycelia and immature chlamydospores), outer layer (chlamydospores) and the glume from the inside out. Ustiloxins concentrations were the highest in the middle layer, followed by the inner layer, and were barely detected in the glume (Wang X. et al., 2016). It has been shown that the initial accumulation of ustiloxins preceded FSB formation as early as 5-9 days after pathogen inoculation and peaked at early FSB maturation (Wang X. et al., 2016; Hu et al., 2020). Nevertheless, in pure-cultured *U. virens*, ustiloxins were barely detected (Wang et al., 2019). This evidence suggests that ustiloxins play important roles in the pathogen-plant interaction (Sun et al., 2020). The concentration of main ustiloxins in contaminated rice grains ranged from 3.46 to 190.47 μg/g, depending on regions and the RFS incidence, and even up to 1.55 mg/g in FSB (Fu et al., Food control, 2018). *In vitro* tests by animal cells showed that ustiloxins could inhibit mitosis by impeding tubulin assembly at 1mM (~494.24 μg/mL) concentration (Ludueña et al., 1994; Ranaivoson et al., 2012). But the underlying mechanisms of ustiloxin toxicity in crops remain unknown. Previously, ustiloxin extraction and purification have been improved to a great extent (Shan et al., 2013; Shan et al., 2014). The phytotoxicity i.e., inhibition of seed germination by different types of ustiloxins were verified (Zheng et al., 2016; Wang et al., 2017). This may provide an insight into the biological roles and molecular mechanisms of ustiloxins.

Rice seed provide an ideal miniature source-sink system allowing us to explore the potential effects of ustiloxins on source (endosperm) and sink (embryo) separately or integrally. In this study, by combined analysis of phenotype, carbohydrates, transcriptome and metabolome of rice seed germination, we aimed to reveal the physiological effects and molecular mechanisms of ustiloxins on rice seed germination. The results would help in understanding of the effects of ustiloxins on rice growth and in *U. virens*-rice interaction, and provide insight for improvement of RFS resistance.

## Materials and methods

### Plant materials and treatment

UA was used here because it is more concentrated and toxic than other types of ustiloxins (Wang et al., 2017). The isolation and purification of UA was established previously (Shan et al., 2013; Shan et al., 2014). Briefly, ground FSB was incubated with distilled water and filtered with double gauze. The solution was concentrated by rotary evaporator and redissolved with ultrapure water, which was then subjected to repeated column chromatography on macroporous adsorption resin HP-20, ODS-AQ, Sephadex LH-20 and Sepha dex G15 to obtain purified UA.

Zhongjiazao-17, one of the most famous indica varieties in southern China, was used in this study. All seeds were rinsed thoroughly with distilled water after surface sterilization with 20% (v/v) NaOCl for 10 min. Forty budded seeds were selected after water-soaking for 24 h, laid on double filter paper infiltrated with ultrapure water (CK) or UA solution in a 9 cm culture dish. Then the dishes were coated with foil paper and placed for germination in a growth chamber in the dark at 25°C. The concentration of UA was set as 200, 50, and 10 µg/mL according to Wang et al. (2017) and Fu et al. (2018). Each dish was treated as a repeat and 4 replicates were included. After 4 days of germination, the counts and length of germ and radicle were measured. Then the seeds were divided into embryo (including germ and radicle) and endosperm parts for further analysis.

### Sugars extraction and assay

Sugars including starch, sucrose, fructose and glucose were extracted following previous research with modification (Shen et al., 2020; Liang et al., 2021). Briefly, endosperm and embryo tissues were ground to fine power, then weighed and incubated with 80% ethanol at 80°C for 2 h. The supernatant was collected for measurement of sucrose, fructose and glucose. The pellet was digested by diluted hydrochloric acid solution (3 mol L^-1^) and neutralized with sodium hydroxide for starch determination. Starch, sucrose, fructose and glucose were determined with corresponding detection reagent kits (Solarbio, Beijing, China) according to the protocols.

### RNA sequencing and analysis

Based on the phenotype, embryo samples of UA200 and CK with 4 replicates were used for transcriptome analysis. RNA-Seq was performed at Biomaker Technologies (Beijing, China) following previous research (Shen et al., 2020). Briefly, DP441 RNAprep Pure Plant Plus Kit (Tiangen, Beijing, China) was used for total RNA extraction. NanoDrop 2000 (Thermo Fisher Scientific, DE, USA) was used to determine RNA concentration and purity. RNA integrity was assessed by the Nano 6000 Assay Kit of the Agilent Bioanalyzer 2100 system (Agilent Technologies, CA, USA). Sequencing libraries were prepared following the manufacturer’s protocol of NEBNext UltraTM RNA Library Prep Kit for Illumina (NEB, USA) and sequenced on an Illumina platform. Clean data were mapped to the newly released indica rice genome (R498_IGDBv3, MBKbase) sequence by using Hisat2 (Kim et al., 2015; Du et al., 2017). Gene expression was estimated by fragments per kilobase of transcript per million reads (FPKM) as listed in **Supplemental Table S1**.

Differential expression analysis of two groups was performed using the DESeq2. Genes satisfying |log_2_(Fold Change)| >1.0, and *p* < 0.01 were defined as differential expressed genes (DEGs) and subjected to hierarchical clustering and enrichment analysis of Gene Ontology (GO), Clusters of Orthologous Groups (COG) and Kyoto Encyclopedia of Genes and Genomes (KEGG) pathway on the platform BMKCloud (http://www.biocloud.net). All DEGs and corresponding annotations were listed in **Table S2**.

### Metabolomic sequencing and analysis

Metabolite profile was performed for both endosperm and embryo with 6 replicates, respectively. Untargeted metabolome analysis was performed at Biomarker Technologies. In brief, samples were extracted by 1 mL mixture of methanol, acetonitrile and water (2/2/1, v/v/v) with an internal standard (2 mg/L) in 1.5 mL centrifuge tubes. After vortexing for 30 s, samples were ground at 45 Hz for 10 min on ice. Solutions were then incubated at -20°C for 1 h, followed by centrifugation at 12 000 rpm, 4°C for 15 min. A volume of 120 μL supernatant from each tube was collected into vial and subjected to UHPLC-QTOF-MS analysis. Quality control was by pooling 10 μL supernatant from each sample.

Metabolites were measured using UPLC Acquity I-Class PLUS (Waters, MA, USA) with Waters UPLC HSS T3 column (1.8μm 2.1*100 mm) coupled to Waters Xevo G2-XS QTof mass spectrometer. The mobile phase was composed of (A) water (0.1% formic acid) and (B) acetonitrile (0.1% formic acid). Gradient programme was set as: 0 min, 98% A; 0.25 min, 98% A; 10 min, 2% A; 13 min, 2% A; 13.1 min, 98% A; 15 min, 98% A with the flow of 0.4 mL/min. The injection volume was 1 μL. ESI ion source conditions were set as: Capillary voltage = 2 000 V or -1 500 V in positive or negative modes; Source temperature = 150°C; Dry temperature = 500°C and dry flow = 800 L/h.

Raw MS data were collected by Waters MassLynx V4.2 and processed by Progenesis QI and identified by databases of METLIN online, Biomaker self-built and HMDB (https://www.hmdb.ca/). Clustering analysis, principal component analysis (PCA) and orthogonal partial least squares discriminant analysis (OPLS-DA) as well as KEGG were performed using BMKCloud. Metabolites satisfying |log2(Fold Change)| >1.0, *p* < 0.05 and variables determined to be important in the projection (VIP) > 1 was adopted to assess differentially expressed metabolites (DEMs, **Table S3**).

### Statistical analysis and illustration

Data processing was performed in Microsoft Excel 2019. ANOVA analysis was conducted using LSD test (*p* < 0.05) in the IBM SPSS Statistics 20. Illustrations were drawn with Microsoft Excel 2019, Origin 2021 and Adobe Illustrator CS6.

## Results

### Inhibition of rice germination by UA

**Figure 1:**
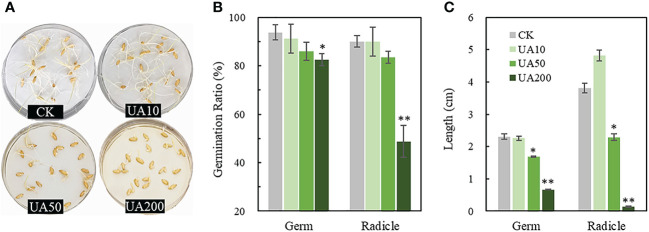
Germination of rice seeds treated with different levels of UA. **(A)** Photograph of germinated seeds; **(B)** Germination ratios and **(C)** Lengths of germ and radicle, respectively. * and ** indicate significant levels at *p* < 0.05 and 0.01, respectively. The unit of 10, 50 and 200 is μg/mL. CK, control check; UA, ustiloxin A.

Inhibition of rice seed germination was evaluated under different UA levels. Both the counts of germinated germ and radicle and the following elongation were reduced, especially under the treatments of UA50 and UA200 (**Figure 1A**). UA10 had almost no effect on seed germination. Besides, the germination ratio was slightly decreased for UA50 than CK. Inhibitory effects of UA200 were 12.0% and 45.8% in germination, 71.0% and 96.5% in elongation for germ and radicle, respectively (**Figures 1B, C**). Swelling of germ and root was also observed.

### Sugar concentration changes in embryo and endosperm

**Figure 2:**
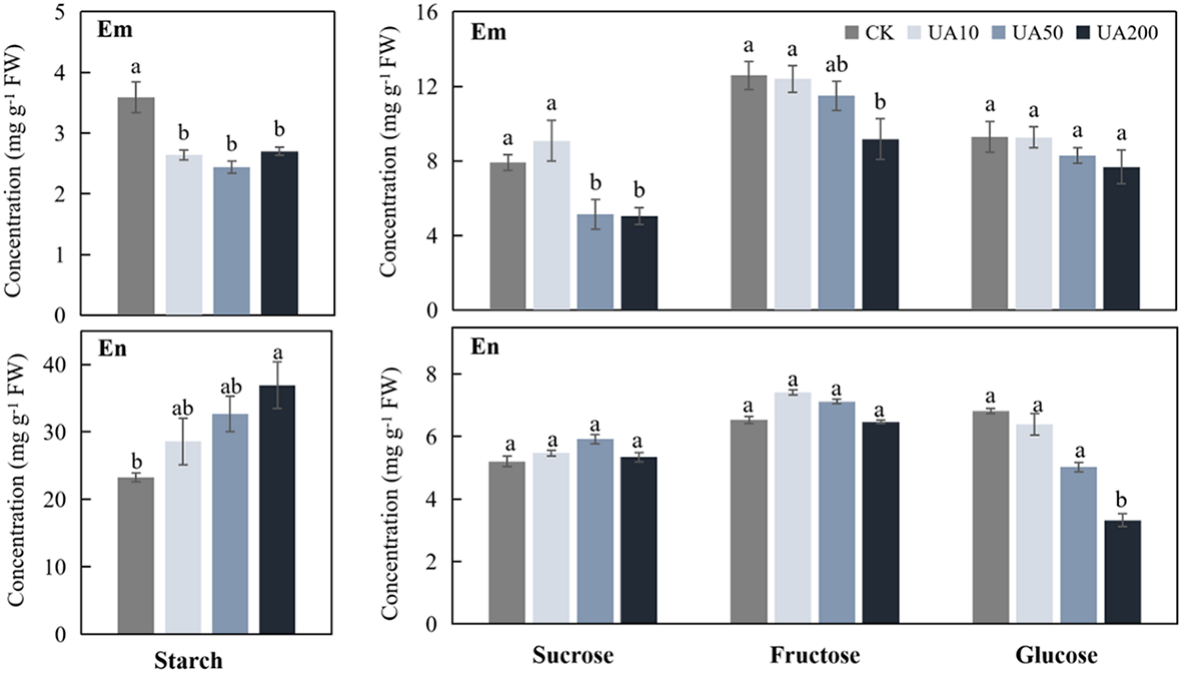
Levels of starch, sucrose, fructose and glucose in embryo (Em) and endosperm (En) under different concentrations of UA treatments. Different letters indicate significant levels at *p* < 0.05.

During seed germination, starch in endosperm is degraded into soluble sugars, which are immediately transported into embryo for consumption. We examined the levels of starch, sucrose, fructose and glucose in both embryo and endosperm under different UA treatments. Starch was much higher in endosperm than in embryo, while the soluble sugars were just the opposite (**Figure 2**). In embryo, both starch and the soluble sugars gradually decreased with the increase of UA concentration (**Figure 2**). When comparing with CK, the starch, sucrose and fructose in UA200, reached significant level of difference (*p* < 0.05) in embryo. So is to the starch and sucrose in UA50. However, the changes of sugars in endosperm were, to some extent, different. Starch in endosperm was higher for all UA treatments than CK especially for UA200. Sucrose and fructose were unchanged but glucose was gradually decreased in endosperm (**Figure 2**).

### Transcriptome analysis of DEGs in embryo

**Figure 3:**
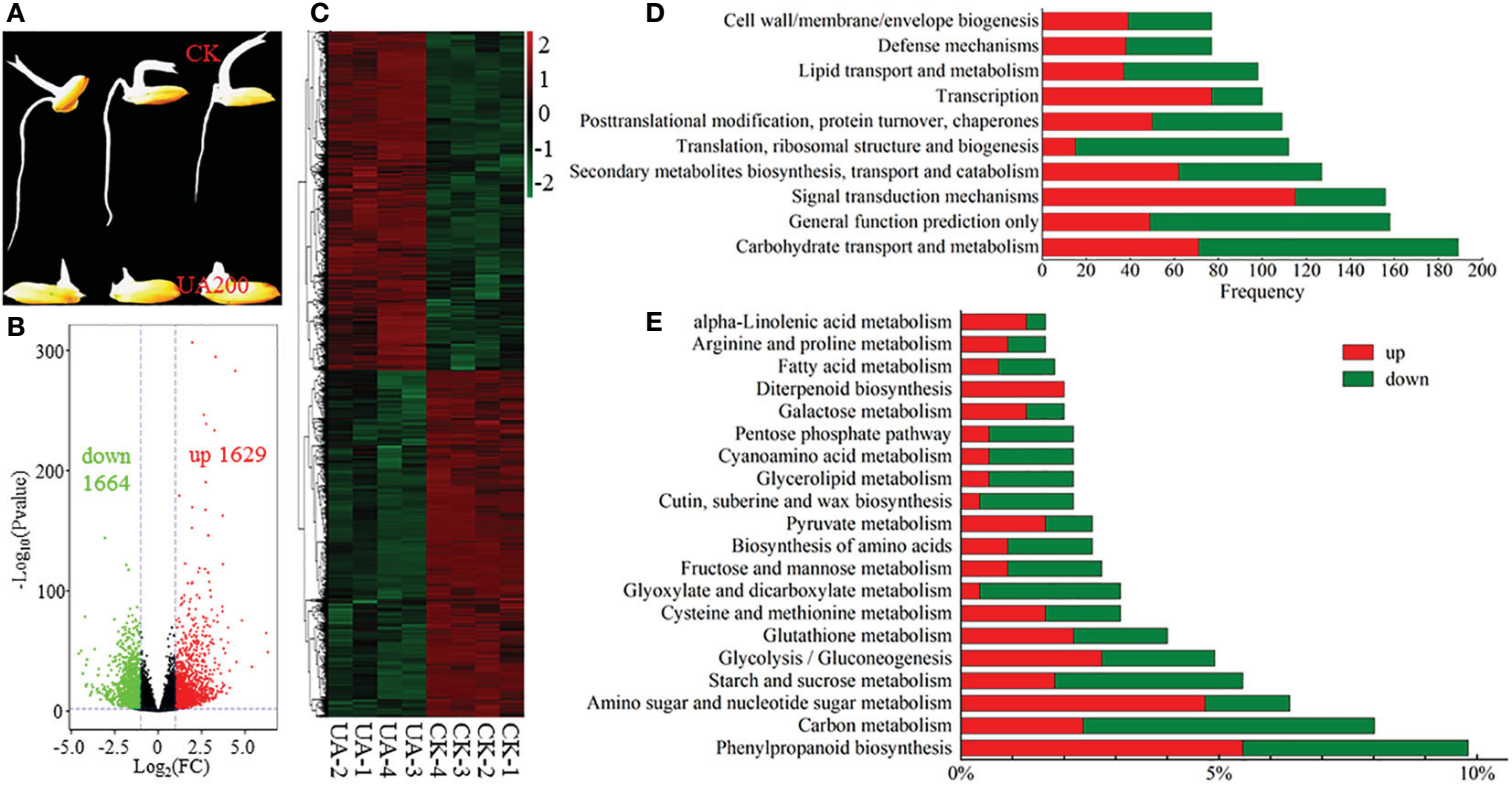
Expression and classification of DEGs. **(A)** Phenotypic contrast between UA200 and CK; **(B)** Volcano plot for the up- and down-regulated DEGs; **(C)** Cluster analysis of all DEGs; **(D)** The top 5 enriched COG function classification of DEGs based on consensus sequence; and **(E)** The top 20 enriched KEGG pathways of DEGs. CK, control check; COG, Clusters of Orthologous Groups; DEGs, differentially expressed genes; KEGG, Kyoto Encyclopedia of Genes and Genomes; UA, ustiloxin A.

RNA-seq was conducted to gain global insight of molecular mechanisms by which UA inhibited embryo growth. The UA200 treatment was chosen because both the germination ratio and coleoptile length reached significant differences compared to CK (**Figures 1B, C**, **3A**). Correlation coefficients within replicates ranged from 0.935 to 0.998, and clear separation between UA and CK by PCA analysis was observed (**Figure S1**). A total of 41594 genes were detected in at least one sample. There were 3293 DEGs, including 1629 genes up-regulated and 1664 down-regulated (**Figure 3B**). The DEGs up or down-regulated clustered into two large groups (**Figure 3C**). COG function classification showed that “carbohydrate transport and metabolism”, “signal transduction mechanisms”, “secondary metabolisms biosynthesis, transport and catabolism” and “lipid transport and metabolism” were highly enriched (**Figure 3D**). The top 20 enriched KEGG pathway of the DEGs are shown in **Figure 3E**. Compared with CK with UA200, the pathway “phenylpropanoid biosynthesis” was the most enriched, followed by “carbon metabolism”, “amino sugar and nucleotide sugar metabolism”, “starch and sucrose metabolism” and “glycolysis/gluconeogenesis”. For most carbon metabolism related pathways such as “starch and sucrose metabolism”, “glycolysis/gluconeogenesis”, “glyoxylate and dicarboxylate metabolism”, “fructose and mannose metabolism” and “pentose phosphate metabolism”, more genes were down-regulated than up-regulated. Similar trends were found in the pathway of “biosynthesis of amino acids” and “fatty acid metabolism”. However, for “pyruvate metabolism”, “galactose metabolism” and “diterpenoid biosynthesis”, there were more genes up-regulated than down-regulated.

### Overview of metabolic profiles and DEMs in embryo and endosperm

**Figure 4:**
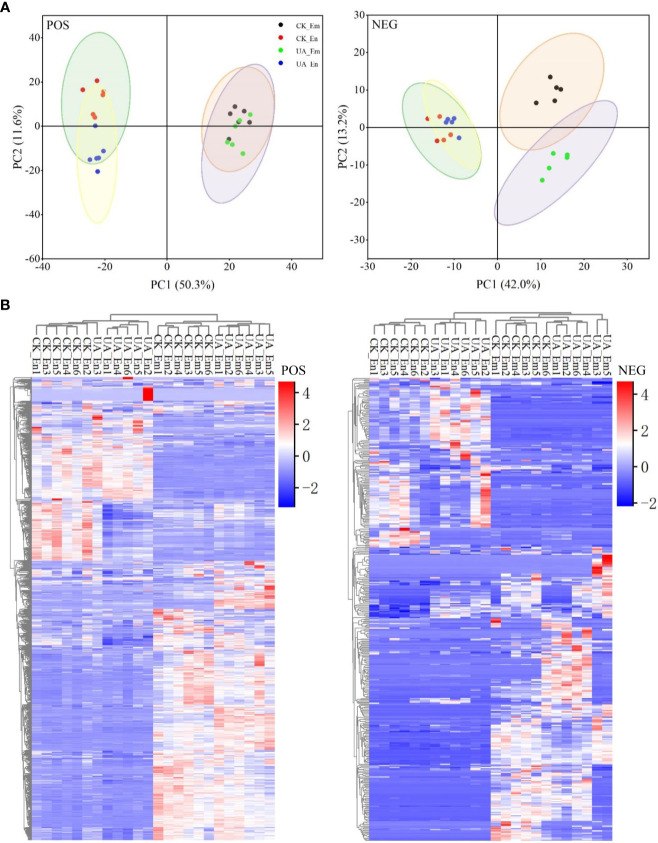
Analyses of principal component **(A)** and cluster heatmap **(B)** for metabolisms of both endosperm (En) and embryo (Em) detected in positive (POS) and negative (NEG) modes, respectively.

To comprehensively uncover the metabolic changes between CK and UA200, a high-throughput untargeted metabolome analysis was performed. A total of 395 metabolites in the negative ionization mode (NEG) and 1083 metabolites in the positive mode (POS) were detected in endosperm (En) and/or embryo (Em). Replicates with correlation values lower than 0.8, i.e. the CK_Em6, UA_En3 and UA_Em2 in the NEG mode were excluded for further analysis (**Figure S2**). PCA scores and heatmap plots in the POS and NEG modes showed that the differences between groups were greatest within organs (En *Vs* Em), followed by treatments (CK *Vs* UA). Metabolites of endosperm in the POS mode and metabolites of embryo in the NEG mode were clearly separated by UA and CK (**Figure 4**). OPLS-DA analysis can effectively remove irrelevant data variation, and the values of Q^2^Y in the models of all contrasts were higher than 0.88, indicating significant separation between the groups (**Figure S2**).

**Figure 5:**
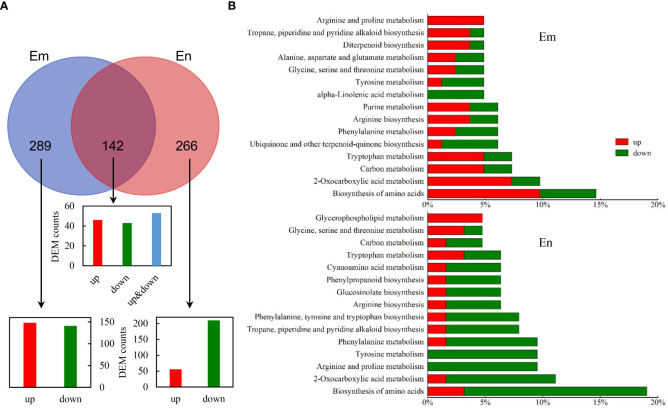
Identification of DEMs in CK_Em vs UA_Em and CK_En vs UA_En. **(A)** Venn analysis of DEMs that up- and/or down-regulated in Em, En and both. **(B)** KEGG annotation of DEMs in Em and En. Abbreviation: DEMs, differential expressed metabolites; Em, embryo; En, endosperm; KEGG, Kyoto Encyclopedia of Genes and Genomes.

DEMs were identified according to VIP > 1 and *p* < 0.05 and were matched with HMDB and KEGG database. For DEMs that detected in both POS and NEG modes, the ones with higher expression abundances and lower *p* values were retained. Finally, we identified 431 (239 up, 192 down) and 408(110 up, 298 down) DEMs in endosperm and embryo, respectively (**Figure 5A** and **Table S3**). 142 DEMs were found in both Em and En, including 46 up-regulated and 43 down-regulated, and 53 inconsistently expressed. Enrichment analysis was performed based on the annotation (**Figure 5B**). In embryo, the DEMs were enriched in amino acid metabolism, carbon metabolism and biosynthesis of secondary metabolites such as alkaloid and diterpenoid. The enriched KEGG pathway of DEMs in endosperm were similar with embryo. Nevertheless, DEMs enriched in the same pathway such as “Biosynthesis of amino acid”, “2-Oxacarboxylic acid metabolism” and “Arginine and proline metabolism” were more down-regulated in endosperm but up-regulated in embryo. “Glycerophospholipid metabolism” in endosperm was up-regulated which indicates lipid peroxidation induced by UA (**Figure 5B**).

### Expressions of SWEET genes are altered by UA

**Figure 6:**
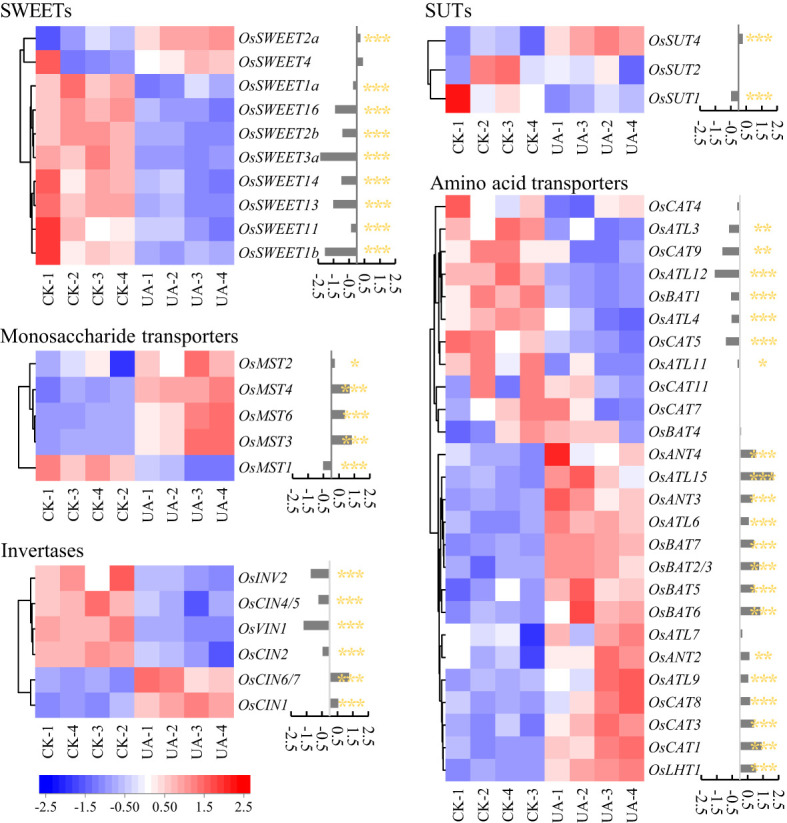
Changes of genes expression related to sugar and amino acid transport between control and UA200. The red and blue represent induced or suppressed, respectively. The *, ** and *** indicate for significant levels at *p* < 0.05, 0.01 and 0.001, respectively. SWEET, sugars will be eventually exported transporter; MST, monosaccharide transporter; INV, invertase; CIN, cell-wall invertase; VIN, vacuolar acid invertase; SUT, sucrose transporter; CAT, cationic amino acid transporter; ATL, amino acid transporter-like; BAT, bi-directional amino acid transporter; ANT, aromatic and neutral amino acid transporter; LHT, lysine and histidine transporter.

Carbohydrates and amino acids are the basis of energy and precursors for metabolic responses to UA. We first sought to explain the differential distribution of carbohydrates between endosperm and embryo. Several classes of genes involved in sugar transport or efflux were detected including sugars will eventually be exported transporters (SWEETs), sucrose transporters (SUTs), monosaccharide transporters (MSTs) and invertases (INVs) (**Figure 6**). Significantly, four SWEET genes (*OsSWEET1b*, *3a*, *13*, *16*) were down-regulated, i.e., |log_2_(Fold Change)| >1.0, and *p* < 0.01. Furthermore, all SWEETs detected in embryo were variously suppressed, except for *OsSWEET2a*, *4* (**Figure 6**). Two MST genes (*OsMST3*, *4*) were up-regulated but the *OsMST1* were suppressed significantly (*p* < 0.001). One cell wall INV (*OsCIN6/7*) was up-regulated while the vacuolar acid invertase (*OsVIN1*) was down-regulated. In addition, *OsSUT1* was also suppressed (*p* < 0.001) by UA200 (**Figure 6**). A total of 26 genes that annotated as amino acid transporters were detected, including 2 down-regulated (*OsATL12*, *OsCAT9*) and 6 up-regulated (*OsLHT1*, *OsCAT1*, *OsBAT2/3*, *OsBAT6*, *OsATL15*, *OsANT4*).

### Glycolysis and pentose phosphate pathways are down-regulated while galactose metabolism is slightly stimulated in embryo

**Figure 7:**
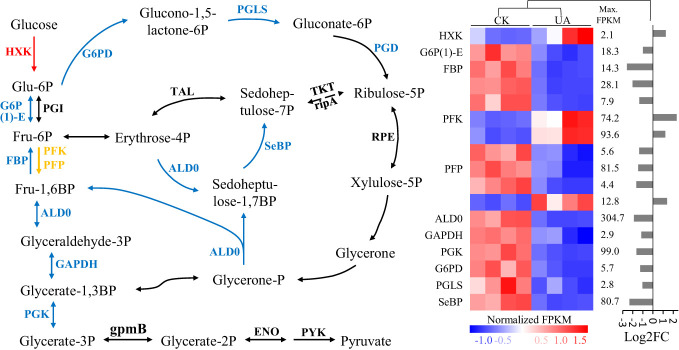
Expression patterns of differentially regulated genes of enzymes involved in glycolysis and pentose phosphate pathways under normal and UA treated conditions. The blue means down-regulated, red means up-regulated, and yellow means up- & down- regulated in **(A)** the pathway; and **(B)** the details of expression for the up- and/or down-regulated genes were shown. HXK, hexokinase; G6P(1)-E, glucose-6-phosphate 1-epimerase; PGI, phosphoglucose isomerase; FBP, fructose-1,6-bisphosphatase I; PFK, 6-phosphofructokinase 1; PFP, diphosphate-dependent phosphofructokinase; ALD0, fructose-bisphosphate aldolase, class I; GAPDH, glyceraldehyde 3-phosphate dehydrogenase; PGK, phosphoglycerate kinase; gpmB, 2,3-bisphosphoglycerate-dependent phosphoglycerate mutase; ENO, enolase; PYK, pyruvate kinase; G6PD, glucose-6-phosphate 1-dehydrogenase; PGLS, 6-phosphogluconolactonase; PGD, 6-phosphogluconate dehydrogenase; TKT, transketolase; rpiA, ribose 5-phosphate isomerase A; TAL, transaldolase; SeBP, sedoheptulose-bisphosphatase; RPE, ribulose-phosphate 3-epimerase; FPKM, fragments per kilobase of transcript per million reads; FC, fold change.

A decrease of available sugars in embryo could be the result of consumption and/or conversion for adaptation to toxin stress. Interestingly, although the only detected two metabolites, glucose and glycerate-2P were unchanged (data not shown), most detected DEGs in the glycolysis and pentose phosphate pathways were down-regulated (**Figure 7** and **Table S2**). The two most changed DEGs, fructose-1,6-bisphosphatase I (FBP) and sedoheptulose-bisphosphatase (SBP) were decreased by 6.3 and 5.2 folds, respectively (**Figure 7**). The DEGs encoding hexokinase (HXK), however, were up-regulated, indicating potentially glucose consumption in other pathways.

**Figure 8:**
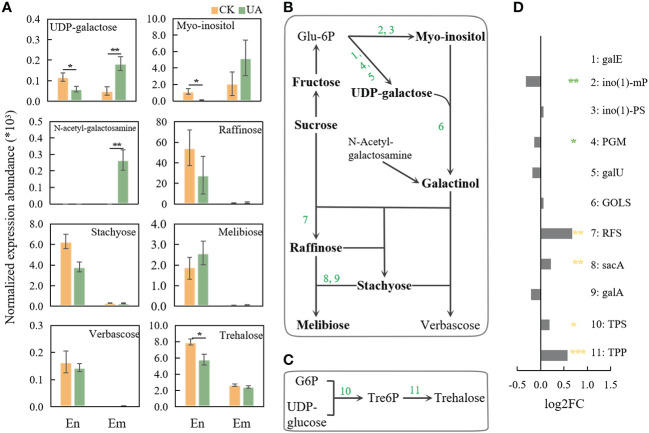
Changes of metabolites and related genes expression of galactose and trehalose pathway. **(A)** Normalized expression abundance of detected metabolites related to galactose metabolism in En and Em. **(B)** The pathway of raffinose metabolism; **(C)** The pathway of trehalose metabolism; **(D)** Changes in gene expression of enzymes related to raffinose and trehalose metabolic pathways after UA treatment compared with the control. The *, ** and *** indicate for significant levels at *p* < 0.05, 0.01 and 0.001, respectively. Abbreviation: galE, UDP-glucose 4-epimerase; ino(1)-mP, myo-inositol-1-monophosphatase; ino(1)-PS, myo-inositol-1-phosphate synthase; PGM, phosphoglucomutase; galU, UTP–glucose-1-phosphate uridylyltransferase; GOLS, inositol 3-alpha-galactosyltransferase; RFS, raffinose synthase; sacA, β-fructofuranosidase; galA, α-galactosidase; TPS, trehalose-6-phosphate synthase; TPP, trehalose-6-phosphate phosphatase.

Galactose metabolism, was reported to be related with stress response (Li X. et al., 2021) and several related metabolites and genes were changed significantly under UA treatment (**Figure 8**). In the embryo, UDP-galactose, as well as N-Acetyl-galactosamine, the precursors for galactinol were up-regulated (**Figures 8A, B**). Myo-inositol, raffinose, stachyose and melibose were slightly induced by UA (**Figure 8A**). Correspondingly, the gene expression of glucosidase (malZ), beta-fructofuranosidase (sacA & INV) and raffinose synthase (RFS) were significantly induced (**Figure 8D**). Several metabolites including UDP-galactose, myo-inositol, N-Acetyl-galactosamine, raffinose and stachyose in the endosperm showed different or opposite response to UA treatment from that in embryo.

Another stress responsive sugar generated from UDPG and G6P, trehalose, was significantly induced in endosperm rather than embryo (**Figures 8A, C**). Accordingly, trehalose-6-phosphate synthase (TPS) and trehalose-6-phosphate phosphatase (TPP) in embryo were significantly induced at different levels (**Figure 8D**).

### Responses of amino acid metabolism to UA stress

**Figure 9:**
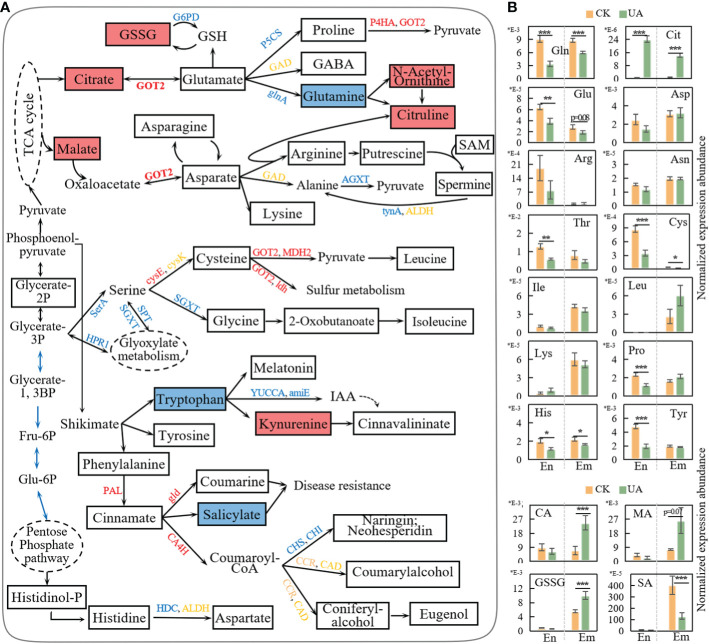
Response of amino acid metabolism to UA treatment. **(A)** simplified representation of metabolism pathways of amino acid and correlated up-and down-stream changes. Genes in red, blue and yellow represent upregulated, downregulated, and both. Rectangle boxes filled with red, blue and white represent upregulated, downregulated and unchanged metabolites, respectively. **(B)** Normalized expression abundance of metabolites related to amino acid metabolism. *, ** and *** indicate for significant levels at *p* < 0.05, 0.01 and 0.001, respectively. amiE, amidase; Arg, arginine; Asn, asparagine; Asp, asparate; CA, citrate; CA4H, cinnamic acid 4-hydroxylase; CHI, chalcone isomerase; CHS, chalcone synthase; Cit, citrulline; Cys, cysteine; G6PD, glucose-6-phosphate 1-dehydrogenase; gld, β-glucosidase; Gln, glutamine; glnA, glutamine synthetase; Glu, glutamate; Gly, glycine; GOT2, aspartate aminotransferase; GSH, glutathione; GSSG, glutathione disulfide; Ile, isoleucine; Leu, leucine; MA, malate; MDH2, malate dehydrogenase; P4HA, prolyl 4-hydroxylase; PAL, phenylalanine ammonia-lyase; Pro, proline; SGXT, L-serine: glyoxylate aminotransferase; Thr, tryptophan; Tyr, tyrosine; YUCCA, indole-3-pyruvate monooxygenase.

Amino acids are central to glycolysis, pentose phosphate pathway and tricarboxylic acid (TCA) cycle, acting as an important source of energy when carbohydrates are scarce. They also act as precursors to numerous secondary metabolites such as alkaloids, organic acids and flavonoids, which are associated with environmental stresses. Here, we detected 15 amino acids including glutamate (Glu), proline (Pro), glutamine (Gln), citrulline (Cit), asparate (Asp), asparagine (Asn), arginine (Arg), cysteine (Cys), leucine (Leu), glycine (Gly), isoleucine (Ile), tryptophan (Thr), tyrosine (Tyr), phenylalanine (Phe) and histidine (His) in endosperm and/or embryo (**Figure 9**). Five amino acids in endosperm (Pro, Glu, Cys, Thr, Tyr) and one in embryo (Glu) were down-regulated (log2FC >1, *p* < 0.05 & VIP >1, **Figure 9A**). Glu, Phe and His in endosperm and Cys, and His in embryo were significantly decreased (*p* < 0.05, **Figure 9B**). The only exception is Cit, which was up-regulated in both endosperm and embryo (**Figure 9**). Several genes involved in the transformation of amino acids were down-regulated such as glutamine synthetase (glnA), converting Glu into Gln, and serine: glyoxylate aminotransferase (SGXT), converting Ser to Gly. In contrast, the expression of aspartate aminotransferase (GOT2) was up-regulated. The corresponding metabolite abundances of citrate and malate were upregulated to approximately 3.5 and 4.0 folds, respectively (**Figure 9A**). The prolyl 4-hydroxylase (P4HA) and malate dehydrogenase (MDH2), responding for the conversion of proline and cysteine to pyruvate, were both upregulated (**Figure 9A**). These, taken together, may imply substrates supplementation of TCA cycle and energy support by amino acids when glycolysis and pentose phosphate pathway are compromised.

Glutathione (GSH) is a key antioxidant that plays a crucial role in plant detoxification and ROS scavenging. Though GSH was not detected, the Glutathione disulfides (GSSG) were significantly upregulated, indicating an increased GSH oxidization. Glutathione reductase can regenerate GSH from GSSG dependent on NADPH. However, the regeneration of NADPH from NADP^+^ was blocked by the down-regulated glucose-6-phosphate 1-dehydrogenase (G6PD) (**Figure 9A**).

Tryptophan, tyrosine and phenylamine are aromatic amino acids derived from the shikimate pathway, which is critical for both plant growth and resistance. Here, two genes (indole-3-pyruvate monooxygenase, YUCCA; amidase, amiE) involved in the synthesis of indoleacetic acid (IAA) were down-regulated, and the abundance of kynurenine was up-regulated (**Figure 9A**). For the phenylpropanoid pathway, gene expression of phenylalanine ammonia-lyase (PAL), cinnamic acid 4-hydroxylase (CA4H) and β-glucosidase (gld) were up-regulated. However, the corresponding downstream products in both endosperm and embryo were unchanged. Instead, the abundances of coumarylalcohol, coniferylalcohol, eugenol, kaempferol and luteolin were slightly depressed by UA (**Figure S3**). This may be due to the activity of CA4H enzyme which is also NADPH-dependent. The expression of chalcone synthase (CHS) and chalcone isomerase (CHI), the two critical genes in flavonoid biosynthesis, were down-regulated (**Figure 9A**). Salicylate (SA), considered as a key stress response substance, was also down-regulated in embryo, and decreased to a lesser extent in endosperm (**Figure 9B**).

### The differential expression abundance of gibberellins in endosperm and embryo

**Figure 10:**
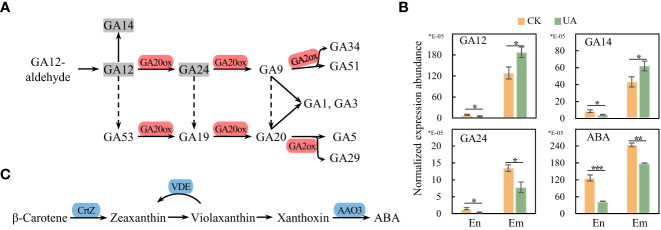
Gene expression and metabolic abundance of GA and ABA synthesis pathways. **(A)** DEGs in the pathway of GAs conversion. **(B)** Normalized metabolic abundances of GAs and ABA in endosperm and embryo. **(C)** DEGs in the ABA synthesis pathway. *, ** and *** indicate for significant levels at *p* < 0.05, 0.01 and 0.001, respectively. GA, gibberellic acid; GA20ox, gibberellin-44 dioxygenase; GA2ox, gibberellin 2beta-dioxygenase; ABA, abscisic acid; CrtZ, β-carotene 3-hydroxylase; VDE, violaxanthin de-epoxidase; AAO3, abscisic-aldehyde oxidase.

Gibberellins (GAs) are reported to be synthesized in embryo and transported to endosperm to promote starch degradation (Wang et al., 2022). In the top enriched KEGG pathway of DEGs, the diterpenoid biosynthesis was up-regulated, consistent with the increased synthesis of GAs in DEMs in embryo (**Figure 10**). Transcripts levels of GA_20_ox and GA_2_ox responsible for transformation of GAs were also up-regulated. Correspondingly, GA12 and GA14 in UA-treated embryo were significantly higher than those in the control (**Figures 10A, B**). Although GA24 was down-regulated under UA treatment, in terms of relative expression abundance, GA24 is only about one twentieth and one tenth of GA12 and GA14, respectively. However, the abundances of both GA12 and GA14 were significantly lower in the UA-treated endosperm than in the control (**Figure 10B**). Abscisic acid (ABA) is another hormone that interacts with GA and affects grain starch decomposition. We found that ABA synthesis was significantly inhibited, and its abundance was down-regulated in both embryo and endosperm (**Figures 10B, C**).

### Downregulation of ribosomal RNAs by UA treatment

**Figure 11:**
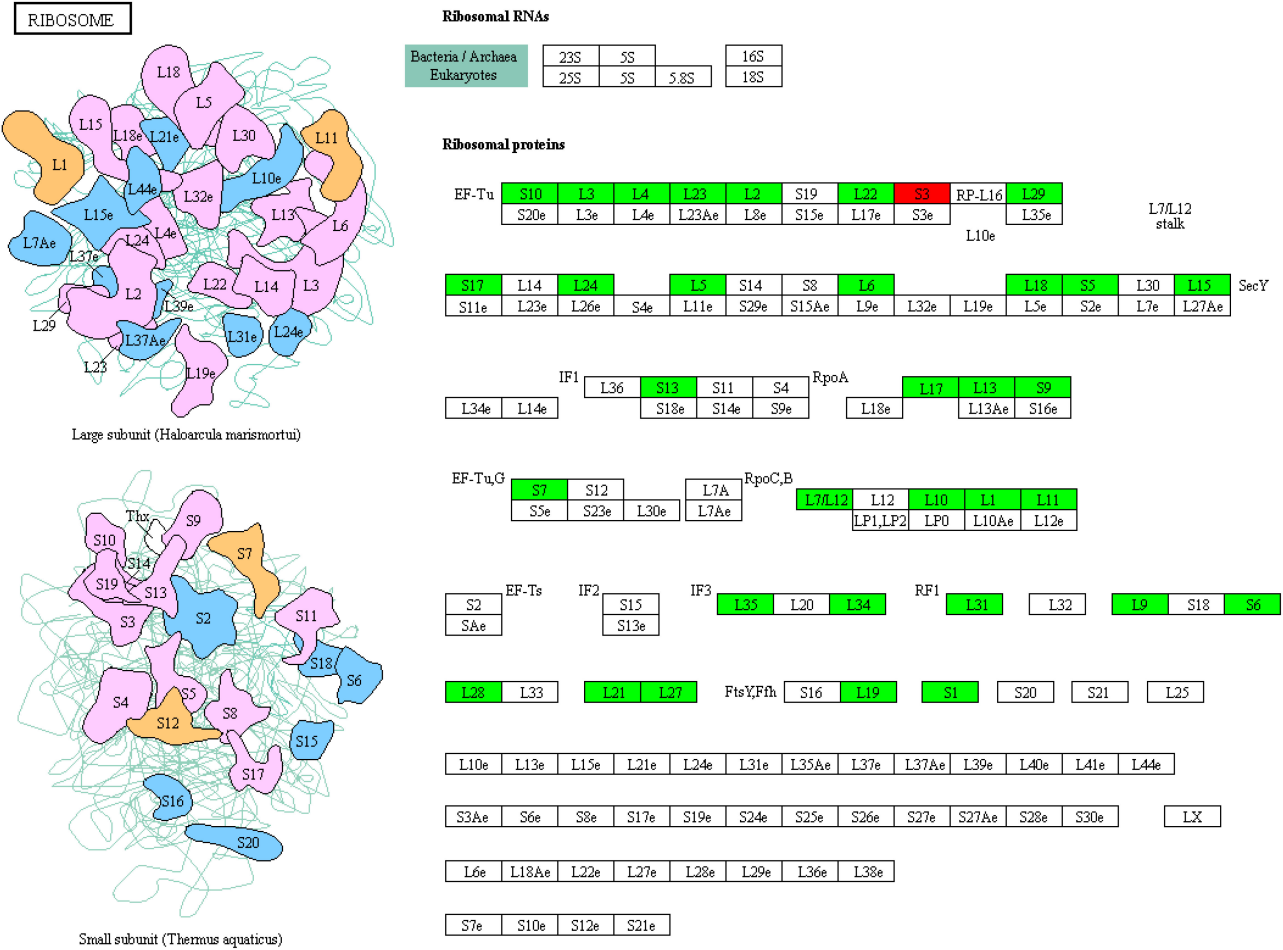
KEGG enrichment of DEGs on ribosome protein synthesis related RNAs.

One of the critical indicators of cellular growth is protein synthesis, which is dependent on the abundance and loading of ribosomes (Sulpice et al., 2014). Our transcriptome analysis showed that the expression of multiple genes of ribosomal RNAs was inhibited by UA, including L1-L7, L9-13, etc. in the large subunit, and S1, S5-6, etc. in the small subunit (**Figure 11**). The only exception was S3, whose expression was up-regulated. These results indicate that UA treatment inhibits cellular protein synthesis and growth of embryo.

## Discussion

### UA toxin inhibits germination by restricting sugars transition and utilization from endosperm to embryo

Rice seeds soaked with ustiloxin A had significantly lower germination counts and growth rates, especially at the concentration of 50 and 200 µg/mL (**Figure 1**; Wang et al., 2017). This inhibition could occur in commercial rice seeds containing FSB, or in rice fields after serious RFS occurrence since ustiloxins are soluble in water. Nevertheless, it is not known by which metabolic processes ustiloxins inhibit germination. Here, for the first time, transcript and metabolite combinations of rice seed germination in response to ustiloxin A were performed. The results of RNA-seq and metabolomics were generally consistent globally. For example, the major enriched KEGG pathways of DEGs included phenylalanine synthesis, carbon metabolism and amino sugar metabolism, etc., while DEMs were most related to amino acid synthesis and carbon metabolism. Furthermore, gene expression was also consistent with changes in key metabolites in specific pathway analyzes including but not limited to amino acid metabolism, raffinose metabolism, and GA metabolism.

Since carbohydrates are the basis of all metabolic activities, we first observed whether carbohydrate supply was normal. The concentrations of starch, sucrose, fructose and glucose were all lower in UA-treated embryo than in controls to different extents. However, the carbohydrates in endosperm showed some opposite trends, suggesting potential problems in sugars transition from endosperm to embryo. The transfer of carbohydrates, mainly sucrose, depends on SUT and SWEET transporter families (Eom et al., 2015; Kim et al., 2021). *OsSWEET3a* has been reported to be involved in glucose transport during rice seedling growth and knockout lines of *OsSWEET3a* showed defects in germination (Morii et al., 2020). *OsSWEET11, 14* and *OsSUT1, 2, 4* were also abundantly expressed in rice seeds at 3 days after germination (Wu et al., 2018). Rice mutations of o*sdof11*, a transcription factor which directly regulates the expression of *OsSWEET11, 14* and *OsSUT1*, are growth-deficient compared with wild-type due to reduced sugar uptake (Wu et al., 2018). Consistent with these findings, our transcriptional analysis showed that *OsSWEET1b, 3a, 13, 16* were down-regulated, and *OsSWEET2b, 11, 14* as well as *OsSUT1* were also significantly decreased (*p* < 0.001) under UA200 treatment. Hence, inhibition of SWEET genes by UA could block the carbon flux from endosperm to embryo and in-turn decrease the sugar availability for growth.

Besides sucrose or glucose, *OsSWEET3a* and the clade III SWEETs (*OsSWEET11, 12, 13,14* and *15*) are also proposed to transmit GA, a pivotal hormone in the activation and breakdown of endosperm starch during germination (Li and Yang, 2020; Morii et al., 2020; Wang et al., 2022; Wu et al., 2022). Since GAs are synthesized in embryo and transported to endosperm to stimulate amylolysis during germination, we further wondered whether the sugar demand signal was efficiently transmitted. Obviously, although the expression of GA_20_ox and GA_2_ox, two critical genes for transformation of different GA forms, and the abundance of GA12 and 14 were up-regulated in embryo, the abundances of GA12, 14 and 24 were much lower in UA-treated endosperm than in CK. The reduction of detected GAs may be not conducive to the decomposition and utilization of starch.

**Figure 12:**
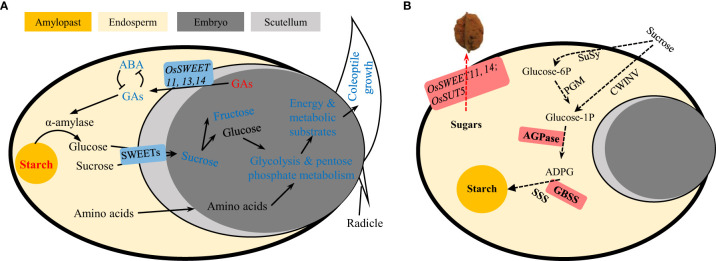
Schematic representation of carbohydrate metabolism during **(A)** seed germination under ustiloxin A treatment and **(B)** grain filling hijacked by U. virens. The red and blue indicate for up- or down-regulated genes or metabolites. GA, gibberellic acid; ABA, abscisic acid; SWEET, sugars will be eventually exported transporter; SUT, sucrose transporter; SuSy, sucrose synthase; PGM, phosphoglucomutase; CIN, cell-wall invertase; ADPG, adenosine diphosphate glucose; AGPase, ADPG pyrophosphorylase; GBSS, granule bound starch synthase; SSS, soluble starch synthase.

Taken together, we propose that the decreased seed germination under UA toxin was, at least partly, due to the blocked sugar transition from endosperm to embryo as well as GAs efflux in the opposite direction by down-regulation of the transporters (*OsSWEET1b, 2b, 3a, 11, 13, 14, 16* and *OsSUT1*). Sugar availability could further influence the subsequent catabolic processes. The down-regulation of genes related to glycolysis and pentose phosphate pathway indicate less metabolism of glucose for generation of energy and substrates (**Figure 12A**).

### Amino acid may act as alternative energy substrates for TCA cycle in UA-treated embryo

Starvation and growth are antagonistic. Ribosomal RNAs, an indicator of protein synthesis and cellular growth in this study were largely down-regulated (**Figure 11**). As substrates for protein synthesis, most of the detected amino acids showed significant or slight decrease in both endosperm and embryo under UA treatment. This is interesting because it has been shown that in many previous studies amino acid levels are increased in stressed environments. For example, proline synthesis and/or arginine metabolism were induced in wheat ears by infection of *Fusarium graminearum* or DON toxin (Warth et al., 2015; Boedi et al., 2016; Duan et al., 2022). When *Salix viminalis* was subjected to phenanthrene stress, or rice filling grain under the stress of 2,2′,4,4′-tetrabromodiphenyl ether, amino acids such as Gln, Glu, Ala, Leu and Tyr were generally elevated (Chen et al., 2019; Li X. et al., 2021). Amino acid accumulation is believed to provide osmotic potential or precursors of secondary metabolites, which are important for crop resistance. However, in this study, one possible speculation is that amino acids were used as substrates for energy metabolism since carbon starvation is a more urgent need than defense. Correspondingly, the expression of GOT2, and the abundances of citrate and malate in TCA cycle were up-regulated (**Figure 9**). Another explanation for the generally decreased amino acids could be variety differences. In a study of rice bacterial leaf blight, the amino acids of disease-resistant strains were generally higher than that of susceptible ones (Sana et al., 2010). Similar trends were also observed in rice leaves infected by sheath rot disease under K^+^ deficiency compared to K^+^ sufficient (Zhang et al., 2021). Although the cultivar (Zhongjiazao-17) used here is high-yielding, stress-resistant and widely adaptable, we do not know its precise resistance to RFS, especially to ustiloxins. Hence, further comparative studies involving multiple varieties are needed in the future.

### UA treatment may inhibit the synthesis of some secondary metabolites

Amino acid metabolism is involved in many plant defense systems like alkaloid and flavonoid synthesis. Derived from phenylalanine, phenylpropanoid metabolism plays key role in responding to stress in plants (Chen et al., 2016; Dong and Linm, 2021; Li J. et al., 2021). PAL is the critical rate-limiting enzyme in the synthesis of phenylpropanoids, which catalyzes the deamination of phenylalanine to produce trans-cinnamate. Here, the PAL gene was significantly up-regulated by UA, so were the downstream genes of CA4H and gld for cinnamate transformation. Nevertheless, the abundance of detected phenylpropanoids including coumarylalcohol, coniferylalcohol, eugenol, kaempferol and luteolin did not increase but slightly decreased. A potential explanation is that the enzymatic activity of CA4H is NADPH-dependent. The regeneration of NADPH from NADP^+^ depends on sufficient carbohydrates for respiration to produce reducing [H+]. The G6PD responsible for NADPH recycle was down-regulated by UA. The same situation also appeared in the glutathione system. GSSG was significantly induced, but the GSH regenerated by NADPH was unchanged. In addition, genes in the IAA synthesis pathway were down-regulated, and SA abundance, which is thought to be involved in plant disease resistance, also decrease significantly. In view of the significant or slight down-regulation of some stress-resistant substances under UA treatment, besides the blocked energy base, another possible explanation is that UA might directly target the synthesis pathways of secondary metabolites. However, further evidence is required to support this claim.

### The effect of UA in seed germination may to some extent, be different from its role in the formation of FSB

The physiological mechanisms by which ustiloxins affect seed germination could help us understand their possible roles in the infection and pathogenicity of *U. virens*. Some opposing results, however, are puzzling. Previous studies have shown that the expression of genes involved in sugar transport in spikelets infected by rice false smut is up-regulated, including *OsSWEET13*, *15* and *OsSUT5.*The AGPase and GBSSII in starch synthesis are also induced, which are considered to be critical for hijacking carbon nutrition by simulating rice grain filling (Chao et al., 2014; Fan et al., 2015). Hence, the roles of UA in seed germination and spikelet infection could be, at least partly contrary. One possible consistent point maybe that raffinose metabolism and trehalose metabolism in seed germination treated with UA are variously induced. Many studies believe that raffinose and trehalose metabolism have resistance to pathogenic microorganisms (Woodcock et al., 2021; MacIntyre et al., 2022; Nunes et al., 2022; Yan et al., 2022). However, stachyose in raffinose metabolism was shown to be a preferential carbon source over sucrose for *U. virens* (Wang Y. Q. et al., 2016). Trehalose is also a direct carbon source for many microorganisms (Ruhal et al., 2013; Vanaporn and Titball, 2020). The metabolites related to the plant’s own resistance to adversity happen to be favoured by the pathogen, which may be the key to the ability of the RFS fungus to parasitize the living body.

There are still many unknowns about the infection and pathogenic mechanism of *U. virens* on rice spikelets, and the specific roles of ustiloxins remains to be explored. How to separate the roles of ustiloxins in pathogen infection is currently extremely difficult. UA injection during rice booting stage provided a way for short-term observation such as 3 h (Hu et al., 2020). But whether the injection effectively imported UA into rice spikelets is unknown. Perhaps it is a feasible method to change the medium toxin combined with pathogen inoculation through *in vitro* culture before the synthesis pathway of ustiloxins was fully elucidated.

In conclusion, for the first time, we performed a comprehensive analysis of transcripts and metabolites responsive of rice seed germination to ustiloxin A. We found an increased starch reserve in endosperm but decreased starch and sucrose in embryo under UA treatments, corresponding to the inhibition of seed germination and growth. The decreased sugar availability is explained by the down-regulation of sugar transport. The shortage of carbon supply is validated by the down-regulated pathways of glycolysis and pentose phosphate, probably leading to amino acid consumption for energy metabolism rather than synthesis of resistant substances. We also found a slightly induced galactose and trehalose metabolism. But as mentioned above, the products such as stachyose and trehalose could be used as carbon source for *U. virens*. Our results provide new insights into the role of ustiloxins on rice growth.

## Data availability statement

The original contributions presented in the study are included in the article/**Supplementary Material**. Further inquiries can be directed to the corresponding authors.

## Author contributions

XF and XGL designed the study. YJ, MY, and XWL performed the experiments. XF and XGL analyzed the data. XF, WP and XGL wrote the paper. MP, RC, YH revised the paper. All authors contributed to the article and approved the submitted version.

## Supplementary material

Supplementary Table 1 All gene expression files.

Supplementary Table 2 Differential expressed genes (DEGs).

Supplementary Table 3 Differential expressed metabolites (DEMs) in endosperm (En) and embryo (Em).

Supplementary Figure 1 Correlation coefficients and PCA analysis for all genes.

Supplementary Figure 2 Correlation coefficients and OPLS-DA analysis for all metabolites.

Supplementary Figure 3 Normalized abundance of several metabolites related to phenylpropanoid metabolism.
