# Supplementary material for: Inhibition of rice germination by ustiloxin A involves alteration in carbon metabolism and amino acid utilization
Source: Front Plant Sci. 2023 May 8;14:1168985. doi: 10.3389/fpls.2023.1168985 (PMC10200953; doi:10.3389/fpls.2023.1168985)
Supplement: Supplementary Figure 1 — Correlation coefficients and PCA analysis for all genes. [file DataSheet_1.pdf]

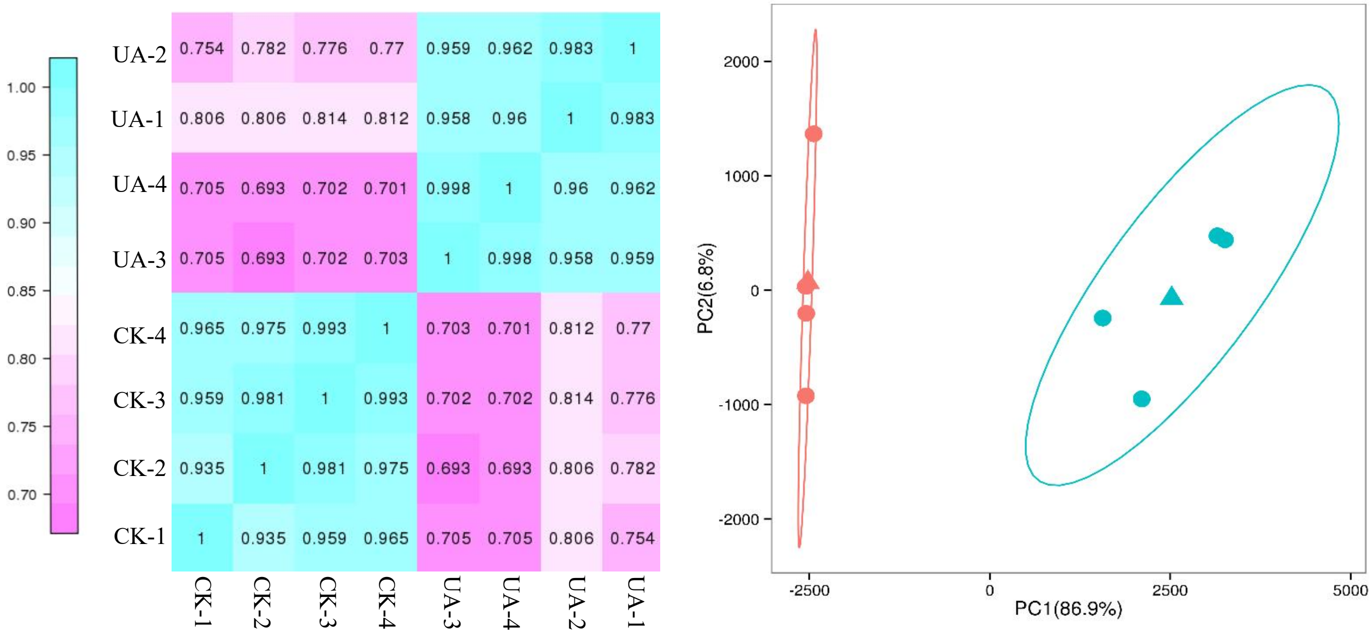

Figure S1. Correlation coefficients and PCA analysis for all genes between ustloxin A and CK treatments.

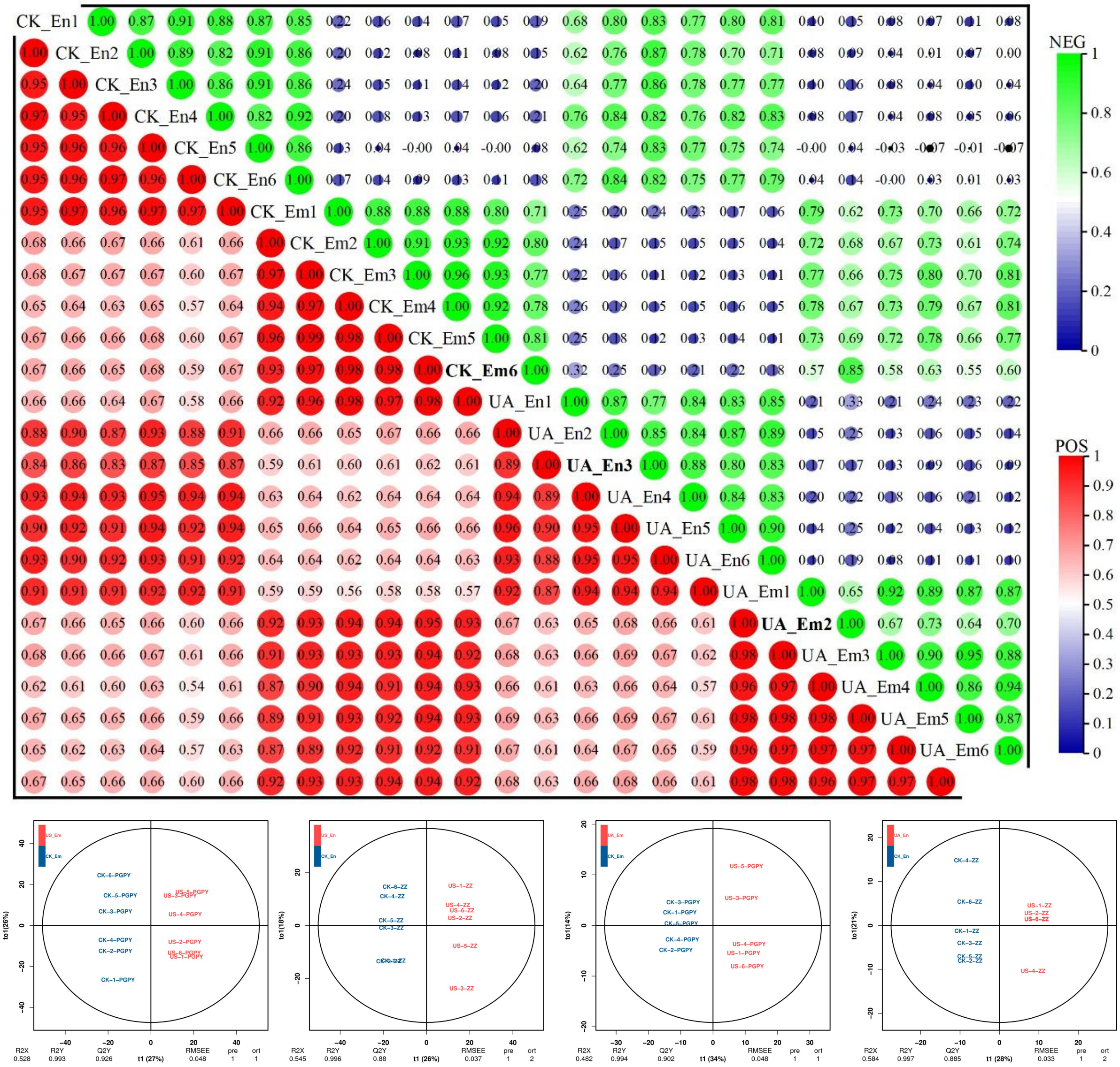

Figure S2. Correlation coefficients and OPLS-DA analysis for all metabolites between ustloxin A and control treatments.

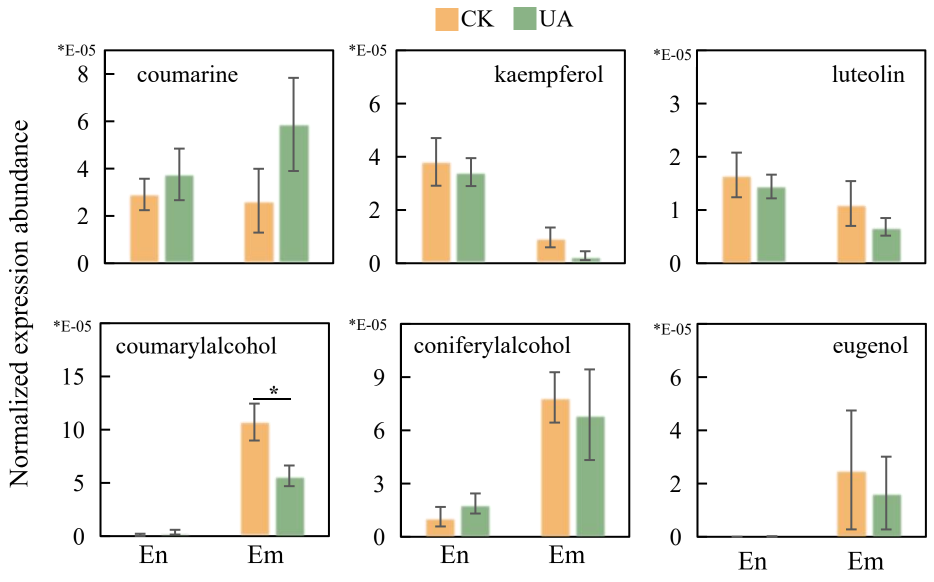

Figure S3. Normalized expression abundance of several detected metabolites related to phenylpropanoid metabolism in endosperm (En) and embryo (Em).
